# Gonadotropins treatment prior to microdissection testicular sperm extraction in non-obstructive azoospermia: a single-center cohort study

**DOI:** 10.1186/s12958-022-00934-1

**Published:** 2022-04-01

**Authors:** Tianwen Peng, Chen Liao, Xin Ye, Zhicong Chen, Yu Lan, Xin Fu, Geng An

**Affiliations:** Department of Obstetrics and Gynecology, Center of Reproductive Medicine, Key Laboratory for Major Obstetric Diseases of Guangdong Province, The Third Affiliated Hospital of Guangzhou Medical University, Guangzhou, 510150 Guangdong China

**Keywords:** Gonadotropins therapy, Non-obstructive azoospermia, Sperm retrieval, ICSI

## Abstract

**Background:**

Microdissection testicular sperm extraction (micro-TESE) in combination with ICSI can make paternity possible for non-obstructive azoospermia (NOA) patients. Testicular sperm can be successfully retrieved in nearly half of NOA patients. Nevertheless, not many convincing protocols are established to improve sperm retrieval rate (SRR). The goal of this study was to evaluate whether gonadotropins therapy before micro-TESE could improve sperm retrieval rate and affect the ICSI outcomes in non-obstructive azoospermia patients with hypergonadotropic hypogonadism.

**Methods:**

This retrospective cohort study included a total of 569 non-obstructive azoospermia men who underwent micro-TESE with or without 3-month of preoperative hCG / hCG plus highly purified urinary FSH (uFSH) between January 2016 and December 2019. The primary outcome was the sperm retrieval rate of micro-TESE.

**Results:**

Sperm was found in 27 patients among 395 NOA men who accepted preoperative gonadotropins treatment (6.8%, 27/395) in post-treatment semen analysis for ICSI. One hundred forty nine out of 542 patients could successfully obtain enough sperm for ICSI through the micro-TESE (overall SRR = 27.5%). There was a statistically significant difference in the SRR between the preoperative gonadotropins treatment and non-gonadotropins treatment groups (31.2%, 115/368 vs. 19.5%, 34/174, *P* = 0.006). In the multivariable analysis with IPTW according to the propensity score, there was a significant association between preoperative gonadotropins treatment and the SRR (OR, 1.59; 95% CI: 1.02–2.52; *P* = 0.042). No differences in the clinical pregnancy rate, live birth delivery rate, or miscarriage rate were observed between the two groups.

**Conclusion:**

Preoperative gonadotropins therapy seems to have a role in improving SRR in NOA patients with hypergonadotropic hypogonadism. We found that gonadotropins therapy had no effect on ICSI clinical outcomes and live birth.

**Supplementary Information:**

The online version contains supplementary material available at 10.1186/s12958-022-00934-1.

## Background

Surgically obtained testicular sperms and intracytoplasmic sperm injection (ICSI) allow non-obstructive azoospermia (NOA) patients to father their genetic offspring [1]. conventional testicular sperm extraction (TESE) or microdissection testicular sperm extraction (micro-TESE) as the gold standard for sperm retrieval in men with NOA reflect their respective advantages. A meta-analysis has suggested that sperm can be found in approximately 50% of NOA patients by surgical sperm retrieval and can result in a live-birth rate (LBR) of up to 28% in ICSI cycle [2]. However, there are currently limited ways to improve sperm retrieval rate (SRR) other than by improving surgeons’ own surgical experience and skills. Until now, whether preoperative endocrine therapy can effectively improve SRR has always been a controversial issue.

Follicle-stimulating hormone (FSH) and luteinizing hormone (LH) are required to regulate testicular development and spermatogenesis [3]. FSH is essential for the initiation of spermatogenesis and is necessary to maintain normal quantitative germ cell production [4, 5]. Evidence suggests that FSH can act to increase spermatogonial differentiation/proliferation [6]. Other studies have shown that FSH stimulates the expression levels of glial cell line-derived neurotrophic factor (GDNF) and fibroblast growth factor 2 (FGF2) in Sertoli cells, which are essential factors for spermatogonial stem cells (SSC) self-renewal and survival [7–9]. Based on the understanding of hormone regulation of spermatogenesis, endocrine therapy has been gradually established in the clinic. Gonadotropin treatment can increase intratesticular testosterone (ITT) levels, which is crucial to promote spermatogenesis [10]. The previous studies had reported the effectiveness of human chorionic gonadotropin (hCG) and human menopausal gonadotropin (hMG) to induce spermatogenesis in azoospermia patients with hypogonadotropic hypogonadism (HH) [11, 12]. However, evidence that gonadotropin therapy could improve SRR in NOA patients with hypergonadotropic hypogonadism is still scant and inconsistent. Hussain et al. [13] reported that the application of hCG with or without hMG before micro-TESE among a subset of patients who did not respond to clomiphene citrate as sole therapy could manifest sperm in the ejaculate in some cases and improve SRR. A multi-institutional prospective study conducted by Shiraishi [14] showed that hCG combined with FSH-based hormonal therapy was effective in a limited sample size of NOA patients before a second micro-TESE attempt. Several other reports [15–17] described NOA patients undergoing endocrine stimulation therapy could be successfully recovered sperm from ejaculated semen. However, a relatively large cohort study did not demonstrate any association between preoperative hormonal therapy (including gonadotrophin injections, selective estrogen receptor modulators, and aromatase inhibitors, etc.) and positive micro-TESE outcomes [18]. No strict criteria were established for which hormone regimen patients received in these studies. Shiraishi et al. [19] conducted another study in a small sample size of patients undergoing secondary surgery but came to the opposite conclusion, found that the effect of gonadotropin therapy seemed to be limited. Our team previously reported that preoperative hCG did not affect SRR or ICSI outcomes of non-mosaic Klinefelter syndrome patients [20]. The effect of endocrine therapy on NOA patients with different etiological classifications is also worth exploring. A further study is required to substantiate this theory.

In this study, we examined whether gonadotropin therapy before micro-TESE can improve sperm retrieval and ICSI outcomes in NOA patients with hypergonadotropic hypogonadism in a relatively large-scale cohort. We also analyzed the correlation of multiple factors, including different ages, infertility duration, body mass index (BMI), types of diagnosis, testicular volume, initial serum total testosterone, and gonadotropins levels.

## Materials and methods

### Study population and treatment protocol

**Fig. 1:**
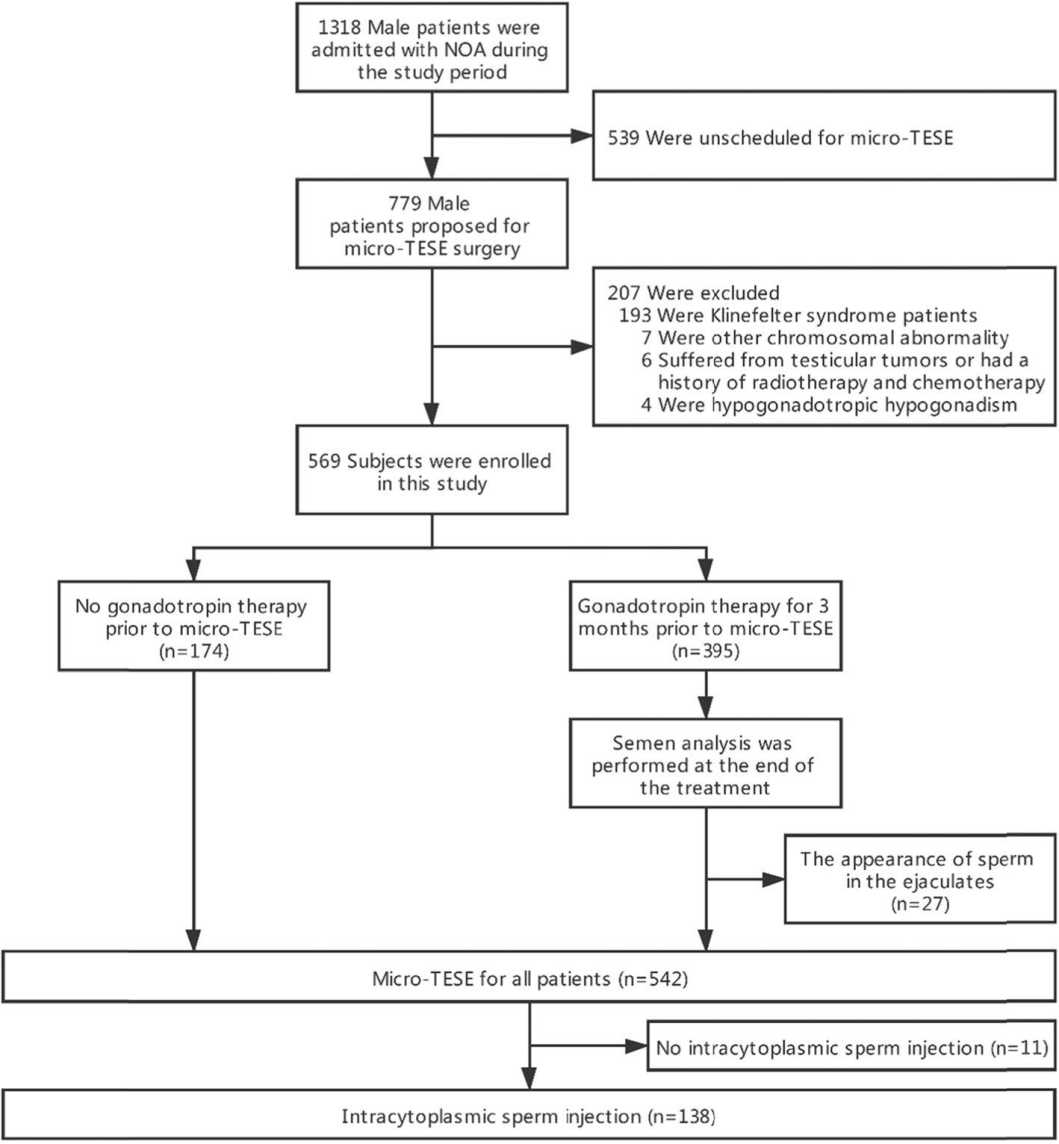
Flow diagram showing study cohort

We retrospectively reviewed the medical records of consecutive NOA patients between January 2016 and December 2019 at the Reproductive Medicine Center of The Third Affiliated Hospital of Guangzhou Medical University. These patients had been required at least two separate occasions for semen analysis. The semen samples of all NOA patients were centrifuged at 3000 g for 30 min to confirm azoospermia. Chromosome testing, AZF gene microdeletion detection, and sex hormone levels were tested. Testicular volume was measured and calculated by ultrasonography. Of the 1318 patients with non-obstructive azoospermia, 779 patients proposed for micro-TESE surgery, and 539 patients received sperm donation for pregnancy. The patients who had Klinefelter syndrome or other chromosomal abnormalities, testicular tumor radiation or chemotherapy, and hypogonadotropic hypogonadism were excluded. A total of 569 NOA patients who proposed for micro-TESE were included in the study (Fig. 1).

All NOA patients had the option of receiving gonadotropins therapy before surgical sperm retrieval without any recommendations regarding possible benefits. One hundred seventy-four patients did not agree to accept any form of preoperative gonadotropins therapy, and 395 patients received gonadotropins therapy without any oral medications for 3 months before surgery. These patients received an intramuscular injection of hCG of 2000 IU every 2 days in the first month. We adjusted the treatment plan according to the changes in serum FSH levels of patients. If serum FSH level did not fall into the normal range (0.7u/L to 11.1 u/L), we would continue to apply hCG for another month before micro-TESE. If serum FSH level was within the normal range, patients would receive alternating injections of 150iu highly purified urinary FSH (uFSH) and hCG every 2 days and sex hormone testing every month for 2 months before micro-TESE. Semen analysis was performed in all NOA patients 3 months after gonadotropins therapy. Sperm was found in semen, and subsequent ICSI was successfully completed in 27 patients. No sperm was found in 368 patients, and then micro-TESE was performed.

### Micro-TESE and cryopreservation of testicular sperm

As described previously, microdissection testicular sperm extraction and cryopreservation of testicular spermatozoa were performed [20]. Briefly, micro-TESE was performed under general anesthesia by a median raphe incision. Testis and epididymis were fully visible. After the albuginea incision, seminiferous tubules were examined under a microscope at 20 to 25 fold magnification. The testicular tissue was minced in G-IVF-Plus (Vitrolife). After a series of treatments, The testicular tissue suspension with sperm was stored and preserved in liquid nitrogen.

### Ovarian stimulation and ICSI

Ovarian stimulation was performed with the use of either GnRH-antagonist or down-regulation protocol with recombinant FSH (Gonal-F, Merck-Serono) [21]. Oocyte retrieval was performed 34–36 h after hCG administration with transvaginal ultrasound. Thawed and fresh testicular sperm were selected for ICSI. The ICSI procedure was performed as described in detail elsewhere [22].

### Embryonic development

Embryo cleavage rate and quality were initially evaluated 44 h after ICSI according to cell number and morphologic parameters. Embryos were scored based on the Society for Assisted Reproductive Technology (SART) scoring system. Embryo transfer was performed on day 3. All couples had at least one embryo to be transferred. Chemical pregnancy was confirmed by assessing serum hCG level 14 days after embryo transfer. Clinical pregnancy was defined by the presence of a gestational sac on a 5-week ultrasound. Live birth was confirmed mainly through telephone interviews with the couple.

### Statistical analysis

The statistical analyses were performed using R software, version 4.0.5 (R Project for Statistical Computing). Two-sided *P* < 0.05 was considered statistically significant. The data were expressed as the median and interquartile range for non-normal distribution continuous variables and numbers and percentages for categorical variables. Baseline characteristics between the treated and untreated groups were compared using Wilcoxon rank-sum test or chi-square test, where appropriate. Multiple imputations were used to handle missing data under the assumption that data were missing at random (MAR).

Crude analysis and multivariable logistic regression models with the use of three propensity score methods, included weighting by inverse probability of treatment weights (IPTW), propensity score matching, and propensity score as an additional covariate, were performed to assess the associations between gonadotropins therapy and SRR. We explored the potential nonlinear associations between SRR and grouping variables (male age, infertility duration, BMI, testicular volume, diagnosis, FSH, LH, and T) using 3-knotted restricted cubic spline regression for determining grouping values. We then performed multivariable logistic analysis stratified by different subgroups. *P* values for interaction were evaluated using interaction terms and likelihood ratio tests. We used the relative excess risk due to interaction (RERI) and corresponding 95% confidence intervals as the measure of interaction on the additive scale. We also used Causal mediation analysis (CMA) to characterize the causality relationship between the primary outcome and different subgroups (diagnosis, male age, BMI, and testicular volume groups). The CDR survival functions were calculated using the Kaplan–Meier method and non-censored values. The ‘time’ response in the model was the order of ETs; the patient was the observational unit, whereas live birth delivery was the event. ICSI outcomes were represented as percentages and compared between 2 groups by Chi-squared test or Fisher’s exact test.

## Results

### Characteristics of the cohort

All the patients were diagnosed with NOA, and the patient characteristics of the gonadotropin therapy and no gonadotropin therapy groups in this study are listed in Table 1. Of the 542 patients, the median age was 31.0 (IQR was [28.0, 34.0]) years old. The composition of NOA causes was different between the two groups and was statistically significant. There was no statistically significant difference in duration of infertility, BMI, bilateral testicular volume, and the serum levels of sex hormones except PRL between the gonadotropins therapy group and non-gonadotropins therapy group. Notably, treatment with either hCG or hCG plus uFSH increased serum testosterone levels. (Supplemental Fig. 1).

**Table 1:** Characteristics of NOA men receiving or not receiving gonadotropins therapy

Characteristic	Overall (*n* = 542)	No GN treatment (*n* = 174)	GN treatment (*n* = 368)	*P*-value
Age (years) (median [IQR])	31.00 [28.00, 34.00]	32.00 [28.00, 35.00]	30.00 [28.00, 33.00]	**0.002**
Duration of infertility, (years) (median [IQR])	3.00 [2.00, 5.00]	3.00 [2.00, 5.00]	3.00 [2.00, 5.00]	0.418
BMI (kg/m^2^) (median [IQR]) ^*a*^	22.00 [20.80, 24.80]	21.80 [20.80, 24.30]	22.30 [20.80, 25.00]	0.529
Bilateral testicular volume (mL) (median [IQR])	6.20 [5.12, 7.90]	6.10 [5.10, 7.80]	6.20 [5.20, 8.10]	0.194
Diagnosis (%)				**0.001**
Idiopathic	396 (73.1%)	146 (83.9%)	250 (67.9%)	
Cryptorchidism	57 (10.5%)	8 (4.6%)	49 (13.3%)	
Previous mumps and bilateral orchitis	51 (9.4%)	10 (5.7%)	41 (11.1%)	
AZFc microdeletion	38 (7.0%)	10 (5.7%)	28 (7.6%)	
Baseline hormone levels (median [IQR]) ^*b*^				
LH (μ/L)	7.03 [4.78, 9.94]	6.95 [4.68, 10.11]	7.08 [4.89, 9.93]	0.730
FSH (μ/L)	18.08 [12.60, 24.53]	18.39 [12.30, 24.68]	17.98 [12.69, 24.25]	0.763
T (nmol/L)	13.61 [9.21, 18.70]	13.66 [8.83, 19.63]	13.59 [9.48, 17.96]	0.622
E_2_ (pmol/L)	80.00 [58.75, 108.25]	80.00 [55.75, 109.50]	81.00 [59.75, 108.00]	0.883
PRL (ng/ml)	9.69 [7.34, 13.21]	8.99 [6.76, 12.33]	10.00 [7.70, 13.40]	**0.028**
Sperm retrieval (%)	149 (27.5%)	34 (19.5%)	115 (31.2%)	**0.006**

Abbreviations: *GN* gonadotropins, *BMI* body mass index, *FSH* follicle-stimulating hormone, *LH* luteinizing hormone, *T* testosterone, *E2* estradiol, *PRL* prolactin, *IQR* interquartile range

^*a*^ The body-mass index is the weight in kilograms divided by the square of the height in meters

^*b*^ Data on the LH level was missing for 20 patients, on the FSH level for 20, on the T level for 37, on the Estradiol level for 46, on the PRL level for 63

The distribution of characteristics of the propensity score-matched samples is also shown in Supplemental Table 1. The odds ratios (with 95% confidence intervals) for the gonadotropins therapy group according to all the variables included in the propensity score model are shown in Supplemental Fig. 2. The C-statistic of the propensity score model was 0.615. In the propensity-matched sample, 174 patients received gonadotropins therapy, and 174 did not. The differences between gonadotropins therapy and baseline variables were attenuated in the propensity score-matched samples as compared with the unmatched samples (Supplemental Fig. 3).

### Sperm retrieval outcomes

Of the 569 patients enrolled in this study, 395 were in the GN treatment group and 174 were not. However, 27 patients (6.8%, 27/395) were treated with ICSI after 3 months of GN therapy with sperm in ejaculated semen (AZFc microdeletion = 55.6%, 15/27; idiopathic = 29.6%, 8/27; orchitis = 7.4%, 2/27, and cryptorchidism = 7.4%, 2/27). Finally, 149 out of 542 patients (Idiopathic = 53.0%, 79/149; AZFc microdeletion = 12.1%, 18/149; orchitis = 19.5%, 29/149) can successfully obtain enough sperm for ICSI through the micro-TESE (SRR = 27.5%).

It was worth noting that there was a statistically significant difference in the SRR between the preoperative gonadotropins therapy and non-gonadotropins therapy groups (31.2% vs. 19.5%, *P* = 0.006, as shown in Table 1). In the crude analysis, patients who had received preoperative gonadotropins therapy were more likely to obtain sperm than those who did not (OR, 1.87; 95% CI: 1.22 to 2.92, *P* = 0.004) (Table 2). In the primary multivariable analysis, inverse probability of treatment weighting was used to adjust for confounding factors between the groups, and the results remained similar (OR, 1.59; 95% CI: 1.02–2.52; *P* = 0.042) (Table 2). After the IPTW, all baseline characteristics were considered balanced, as evidenced by all pairwise and average SMDs of < 0.10 (Supplemental Fig. 2). Multivariable regression analysis with a propensity score-matched sample yielded similar results (OR, 1.80; 95%CI: 1.04–3.16; *P* = 0.037) (Table 2). Both multivariate logistic regression models adjusted for propensity score, multivariable model 1, multivariable model 2, and the full multivariate logistic regression models showed that preoperative gonadotropin therapy remained significantly associated with the successful sperm retrieval outcome (Table 2).

**Table 2:**
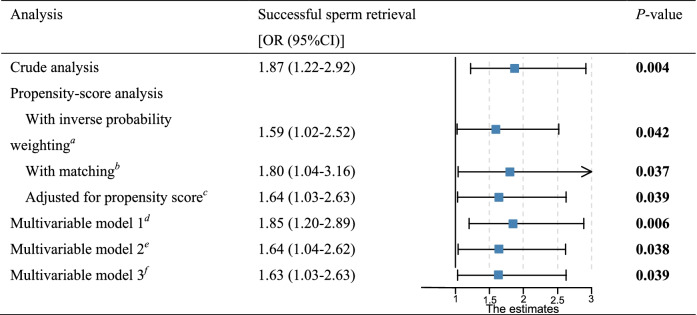
Associations between gonadotropins therapy and the SRR in the crude analysis, propensity-score analyses, and multivariable analysis

^*a*^ Shown is the analysis with an odds ratio from the multivariable Logistic regression model with adjustment for age, duration of infertility, body mass index, diagnosis, baseline LH, baseline FSH, baseline T, baseline E_2_, and baseline PRL with inverse probability weighting according to the propensity score. The analysis included all the 542 patients

^*b*^ Shown is the odds ratio from the multivariable Logistic regression model with the same strata and covariates with matching according to the propensity score. The analysis included 348 patients (174 who received preoperative gonadotropin therapy and 174 who did not)

^*c*^ Shown is the odds ratio from the multivariable Logistic regression model with the same strata and covariates, with additional adjustment for the propensity score. The analysis included all the patients

^*d*^ Shown is the odds ratio from the multivariable Logistic regression model with adjustment for age (model 1). The analysis included all the patients

^*e*^ Multivariable model 2 was further adjusted for duration of infertility, body mass index, and diagnosis. The analysis included all the patients

^*f*^ Multivariable model 3 was further adjusted for sex hormones levels. The analysis included all the patients

### Subgroup analyses and causal mediation analyses of sperm retrieval outcomes

Subsequently, we conducted subgroup analyses to assess the relationship between preoperative gonadotropin therapy and successful sperm retrieval outcomes in different subgroups (Table 3). We explored the potential nonlinear associations between SRR and grouping variables using 3-knotted restricted cubic spline regression for determining grouping values. (Supplemental Fig. 4). The results showed that the application of preoperative gonadotropin treatment in different subgroups of age, duration of male infertility, BMI, mean bilateral testicular volume, causes of NOA, and baseline sex hormone levels resulted in better sperm retrieval outcomes consistently. In particular, patients had higher success rates of sperm acquisition after the administration of gonadotropins therapy in the subgroup with younger age (< 35 years), longer infertility duration (> 3 years), higher BMI (> 24 kg/m2), larger testicular volume (> 6 mL), causes except for the idiopathic NOA, and higher baseline sex hormones levels. The association between gonadotropins therapy and SRR was more pronounced among participants with longer infertility duration (> 3 years) (adjusted OR, 2.85; 95%CI, 1.39–5.84), compared with those with shorter the 3 years (adjusted OR, 1.08; 95%CI, 0.59–1.99) (*P* = 0.041 for interaction). No statistically significant differences were seen in the measure of interaction on the additive scale. CMA showed that gonadotropins therapy mediated 7.4% (*P* = 0.01) of the beneficial effect of non-idiopathic NOA (*P* = 0.04 for ACME) in terms of sperm retrieval outcomes (Supplemental Fig. 5).

**Table 3:**
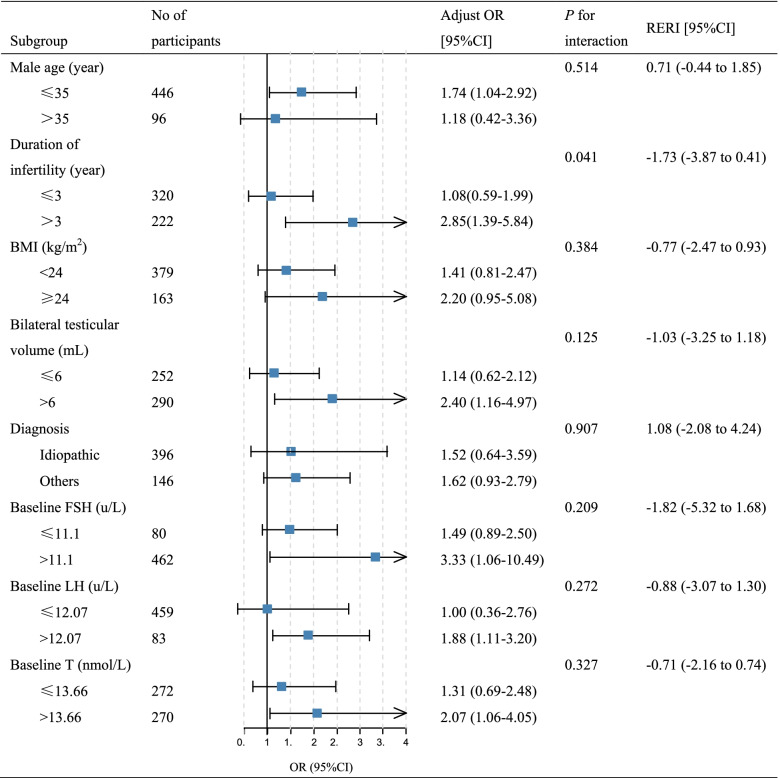
Subgroup analyses of the associations between preoperative gonadotropin therapy and successful sperm retrieval outcome stratified by different factors

Abbreviations: *BMI* Body mass index, *FSH* Follicle-stimulating hormone, *LH* Luteinizing hormone, *T* Testosterone, *OR* Odds ratio, *RERI* Relative excess risk

### ICSI outcomes

Sperms were surgically retrieved in 149 patients, but only 138 patients underwent ICSI treatment. (Reasons for the 11 patients who did not undergo ICSI, including divorce, not yet married, fertility preservation only, other personal reasons). A total of 68 patients (49.3%) achieved a live birth delivery after one or more ET(s) from one cycle. The CDR survival plots show that CDR was higher in preoperative gonadotropins therapy than in the non-gonadotropins therapy group, but the difference was not statistically significant (50.5% vs. 45.2%, *P* = 0.55; Supplemental Fig. 6). There was no significant difference between the two groups for the 2PN cleavage rate, D3 available embryos rate, blastocyst formation rate, β-hCG positive rate, clinical pregnancy rate, live birth delivery rate, and miscarriage rate (Supplemental Table 2).

## Discussion

Spermatogenesis is a complex and dynamic developmental process that includes stem cell proliferation and differentiation, meiotic cell divisions, and extreme chromatin condensation, as well as the regulation of sex hormones. Relevant studies show that LH indirectly stimulates spermatogenesis via intratesticular testosterone (ITT) [23], whereas FSH acts directly on the seminiferous tubules [24]. High ITT is a critical step at the end of spermatogenesis to promote the differentiation of spermatids into mature spermatozoa [25, 26]. FSH plays a crucial role in the initiation and maintenance of spermatogenesis by stimulating Sertoli cell maturation, stimulating the production of androgen-binding protein (ABP), which acts to maintain high levels of ITT, spermatogonial division, and possibly an anti-apoptotic action on spermatogonia and spermatocytes [10, 27, 28]. Previously, administration of exogenous hCG and FSH has been classically found to effectively induce spermatogenesis for non-obstructive azoospermia in men with hypogonadotropic hypogonadism [11, 12]. However, the use of gonadotropins therapy in other etiological types of NOA with hypergonadotropic hypogonadism, especially for idiopathic etiology, remains controversial.

In this study, our results showed that gonadotropins therapy with hCG alone or combined uFSH could treat some NOA patients to find sperm in ejaculated semen and improve SRR of micro-TESE. The effect of gonadotropins therapy was consistent in subgroups of clinical factors, including age, BMI, testicular volume, types of diagnosis, initial serum total testosterone, and gonadotropin levels. In addition, gonadotropins therapy was not related to CDR per the ICSI cycle. Sertoli cells can maintain hormonal responsiveness during FSH stimulation as FSH receptors recycle. However, elevated testosterone levels cause a reduction of serum FSH and LH in NOA men after hCG treatment through negative feedback on the hypothalamic-pituitary-gonadal axis, allowing the Sertoli cells to rest and so allowing the construction of an intratesticular environment of “FSH resetting” [29]. Aydos et al. [30] reported that using pure FSH in NOA patients with normal FSH levels in all histopathological patterns and could improve the success of testicular sperm extraction. hCG hormone therapy has been shown to increase the expression of spermatogonial proliferative nuclear antigen (PCNA) [26, 31]. Examples of effective treatment of oligospermia administrating exogenous gonadotropin stimulation may indirectly prove this inference. Schill et al. [22] reported a 15.3 million/mL increase in mean sperm concentration after 3 months of treatment with hCG and hMG in men with idiopathic oligospermia and normal levels of gonadotropins. We believe that exogenous hormones play an essential role in promoting spermatogenesis.

In our study, 542 NOA patients underwent the first micro-TESE, including 368 patients who completed 3 months of hCG / hCG plus FSH treatment and 174 patients without any hormone therapy, and showed a statistically significant difference in SRR between the gonadotropin therapy group and the non-gonadotropin therapy groups (SRR: 31.2% vs. 19.5%, *P* = 0.006). In the crude analysis, patients who received preoperative gonadotropin treatment had higher SRR than those who did not (OR,1.87; 95% CI: 1.22 to 2.92, *P* = 0.004). Inconsistent treatment effects with gonadotropin therapy may be explained by the bias of related studies [13, 18]. Reifsnyder et al. [18] reported various types of preoperative hormone therapy, including hCG therapy, and found that they did not result in different SRR, possibly due to an imbalance in the number of subjects between the treated and untreated groups. Hussein et al.’s study [13] did not report demographic characteristics, etiologic diagnoses, and baseline sex hormone levels in the treatment and control groups, all of which may be confounding factors that could affect the determination of sperm retrieval outcomes. We conducted various approaches to address the limitations mentioned above, including propensity score weighting, matching, and regression. The IPTW method resulted in the most minor estimated difference between the treated and untreated groups, as evidenced by the SMD results (Supplemental Fig. 3), so this analysis is more stable and accurate in concluding the effectiveness of gonadotropin treatment (OR, 1.59; 95% CI: 1.02–2.52; *P* = 0.042). We also used propensity scores to match 174 pairs of treated and untreated men, and the same conclusions can be drawn (OR, 1.80; 95% CI: 1.04–3.16; *P* = 0.037).

In the subgroup analysis, it is noteworthy that the gonadotropins treatment effect was consistent across different subgroups. This may yield the assumption that men with NOA can improve the first micro-TESE outcomes in most cases following gonadotropin therapy. Additionally, patients had higher SRR after the administration of gonadotropins therapy in the subgroups with younger age (< 35 years), longer infertility duration (> 3 years), higher BMI (> 24 kg/m2), larger testicular volume (> 6 mL), diagnosis except for the idiopathic NOA, and higher baseline sex hormones levels.

The intra-testicular spermatogenic microenvironment may differ in patients with different etiologies of NOA [32], which may lead to differences in the effectiveness of gonadotropins therapy. In our study, the association between gonadotropins therapy and SRR substantially reduced when adjusting for the causes of NOA, although the association remained. We conducted causal mediation analysis and found that patients with a definite cause responded better to gonadotropins compared to the idiopathic azoospermia population. The results showed that gonadotropin therapy mediated 7.4% (*P* = .01) of the beneficial effect of non-idiopathic NOA (*P* = 0.04 for ACME) in terms of SRR.

We also compared the outcomes of ICSI cycles between the gonadotropins treatment or non-gonadotropins treatment group, and we found that there were no significant differences in laboratory parameters, clinical pregnancy rate, CDR, and miscarriage rate, except for the fertilization rate (FR). Furthermore, approximately 7% of patients with successful sperm retrieval had not completed the ICSI cycle for various reasons and had no live-birth outcomes. Although the results were generally consistent with a previous study [18], the hormone treatment regimen was more representative than the present study.

In conclusion, preoperative gonadotropin therapy seems to have a role in improving sperm retrieval rates. The improved outcomes of the first micro-TESE following gonadotropins therapy can be seen in different subgroups. The mediating effect of gonadotropins therapy on types of diagnosis in terms of SRR was noticeable, suggesting that we should apply preoperative gonadotropins therapy in NOA patients more aggressively. In addition, no associations were found between gonadotropins therapy and clinical pregnancy, live birth, and miscarriage outcomes per ICSI cycle. Retrospective analysis rather than randomized controlled trials is a limitation of the present study. Even though we used various methods, including propensity-score methods, to control the confounders, unmeasured factors might have biased our results. More rigorously designed, standardized interventions and larger sample size multicenter randomized controlled trials are needed to provide more substantial evidence-based evidence to verify those results.

## Supplementary Information

**Additional file 1: Supplemental Fig. 1.** Follow-up records of sex hormone levels in hCG (A) and hCG plus uFSH (B) treatment groups.

**Additional file 2: Supplemental Fig. 2.** Odds ratios (95% CIs) of receiving preoperative gonadotropin therapy for all variables included in the propensity score model.

**Additional file 3: Supplemental Fig. 3.** Standardized mean difference (SMD) of variables before and after propensity score matching and weighting.

**Additional file 4: Supplemental Fig. 4.** Association of clinical characteristics with SRR in NOA men.

**Additional file 5: Supplemental Fig. 5.** Causal mediation analysis for gonadotropin therapy.

**Additional file 6: Supplemental Fig. 6.** Cumulative delivery plots and their correspondent tables.

**Additional file 7: Supplemental Table 1.** Characteristics of NOA men receiving or not receiving gonadotropins therapy after the propensity score match.

**Additional file 8: Supplemental Table 2.** ICSI outcomes of couples stratified according to preoperative gonadotropin therapy and non-gonadotropin therapy.

## Data Availability

Some or all data sets generated during and/or analyzed during the present study are not publicly available but will be made available from the corresponding author on reasonable request.

## Abbreviations

Micro-TESE: Microdissection testicular sperm extraction
NOA: Non-obstructive azoospermia
SRR: Sperm retrieval rate
ICSI: Intracytoplasmic sperm injection
LBR: Live-birth rate
uFSH: Highly purified urinary FSH
hCG: Human chorionic gonadotropin
hMG: Human menopausal gonadotropin
ITT: Intratesticular testosterone
HH: Hypogonadotropic hypogonadism
GN: Gonadotropins
BMI: Body mass index
FSH: Follicle-stimulating hormone
LH: Luteinizing hormone
T: Testosterone
E2: Estradiol
PRL: Prolactin
IQR: Interquartile range
IPTW: Inverse probability of treatment weights
RERI: Relative excess risk due to interaction
CMA: Causal mediation analysis
CDR: Cumulative delivery rate
ET: Embryo transfer
OR: Odds Ratio
ACME: Average causal mediation effects
ADE: Average direct effects
βTot: Total effect
βInd: Total indirect
βDir: Direct effect
