# Supplementary material for: Gonadotropins treatment prior to microdissection testicular sperm extraction in non-obstructive azoospermia: a single-center cohort study
Source: Reprod Biol Endocrinol. 2022 Apr 1;20:61. doi: 10.1186/s12958-022-00934-1 (PMC8973804; doi:10.1186/s12958-022-00934-1)
Supplement: Supplementary file 1 — Additional file 1: Supplemental Fig. 1. Follow-up records of sex hormone levels in hCG (A) and hCG plus uFSH (B) treatment groups. [file 12958_2022_934_MOESM1_ESM.docx]

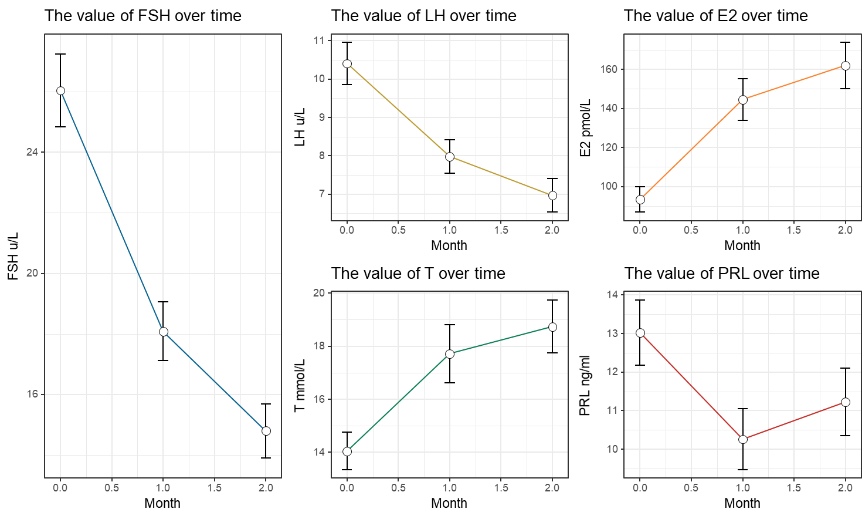


(A)


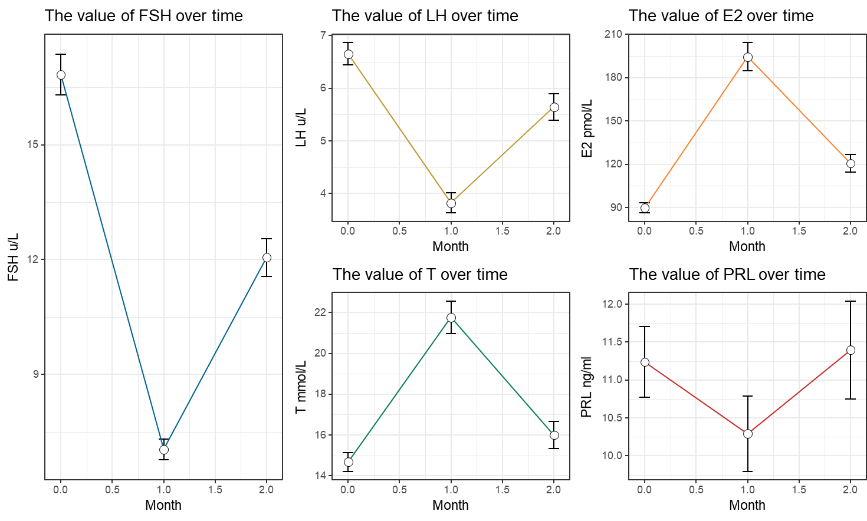


(B)

**Supplemental Fig. 1** Follow-up records of sex hormone levels in hCG (A) and hCG plus uFSH (B) treatment groups.

Abbreviations: FSH: Follicle-stimulating hormone; LH, Luteinizing hormone; T, Testosterone; E_2_, Estradiol; PRL, Prolactin.
