# Supplementary material for: Gonadotropins treatment prior to microdissection testicular sperm extraction in non-obstructive azoospermia: a single-center cohort study
Source: Reprod Biol Endocrinol. 2022 Apr 1;20:61. doi: 10.1186/s12958-022-00934-1 (PMC8973804; doi:10.1186/s12958-022-00934-1)
Supplement: Supplementary file 2 — Additional file 2: Supplemental Fig. 2. Odds ratios (95% CIs) of receiving preoperative gonadotropin therapy for all variables included in the propensity score model. [file 12958_2022_934_MOESM2_ESM.docx]

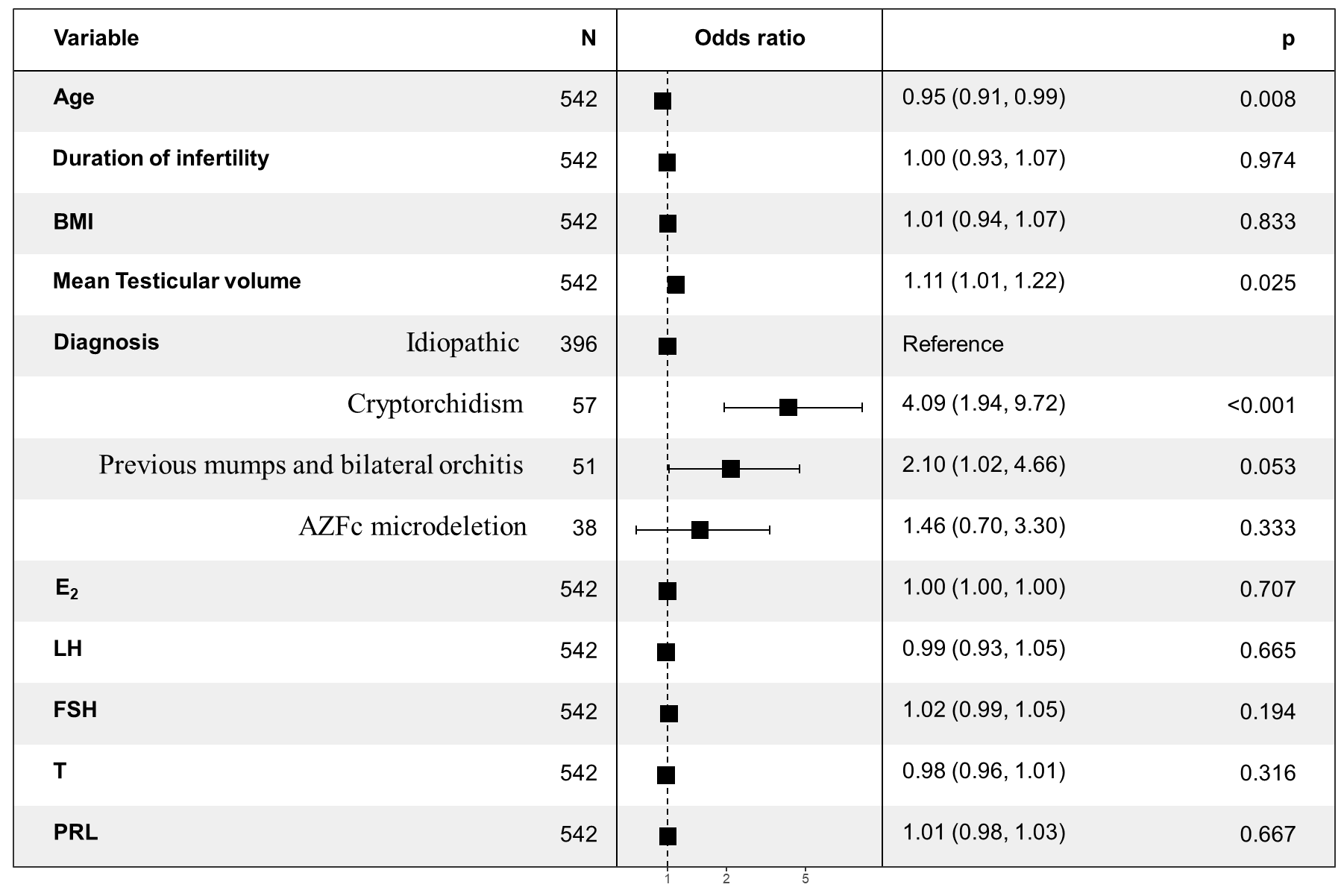


**Supplemental Fig. 2** Odds ratios (95% CIs) of receiving preoperative gonadotropin therapy for all variables included in the propensity score model.
