# Supplementary material for: Gonadotropins treatment prior to microdissection testicular sperm extraction in non-obstructive azoospermia: a single-center cohort study
Source: Reprod Biol Endocrinol. 2022 Apr 1;20:61. doi: 10.1186/s12958-022-00934-1 (PMC8973804; doi:10.1186/s12958-022-00934-1)
Supplement: Supplementary file 3 — Additional file 3: Supplemental Fig. 3. Standardized mean difference (SMD) of variables before and after propensity score matching and weighting. [file 12958_2022_934_MOESM3_ESM.docx]

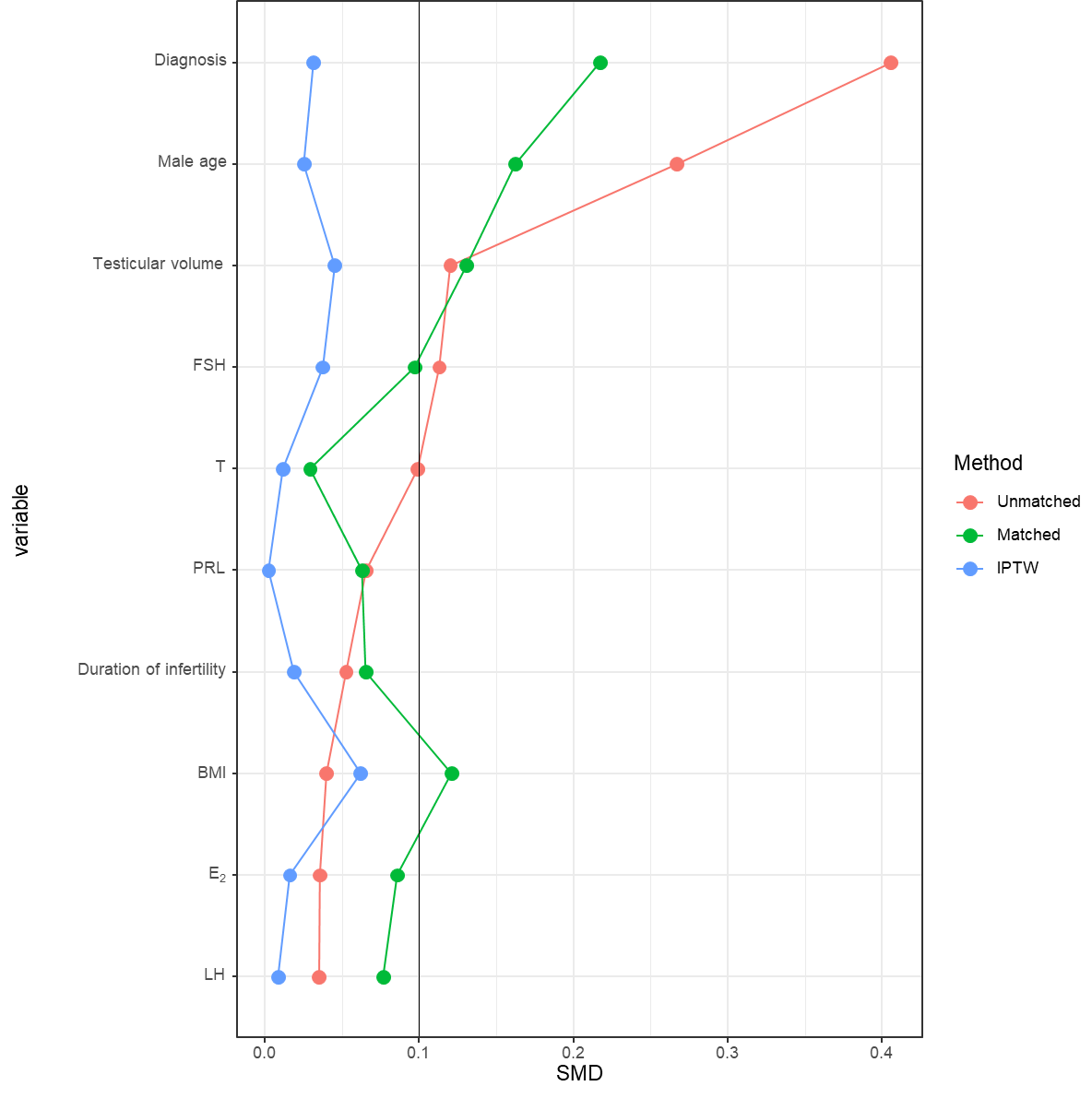


**Supplemental Fig.3** Standardized mean difference (SMD) of variables before and after propensity score matching and weighting.
