# Supplementary figures and images for: Gonadotropins treatment prior to microdissection testicular sperm extraction in non-obstructive azoospermia: a single-center cohort study
Source: Reprod Biol Endocrinol. 2022 Apr 1;20:61. doi: 10.1186/s12958-022-00934-1 (PMC8973804; doi:10.1186/s12958-022-00934-1)

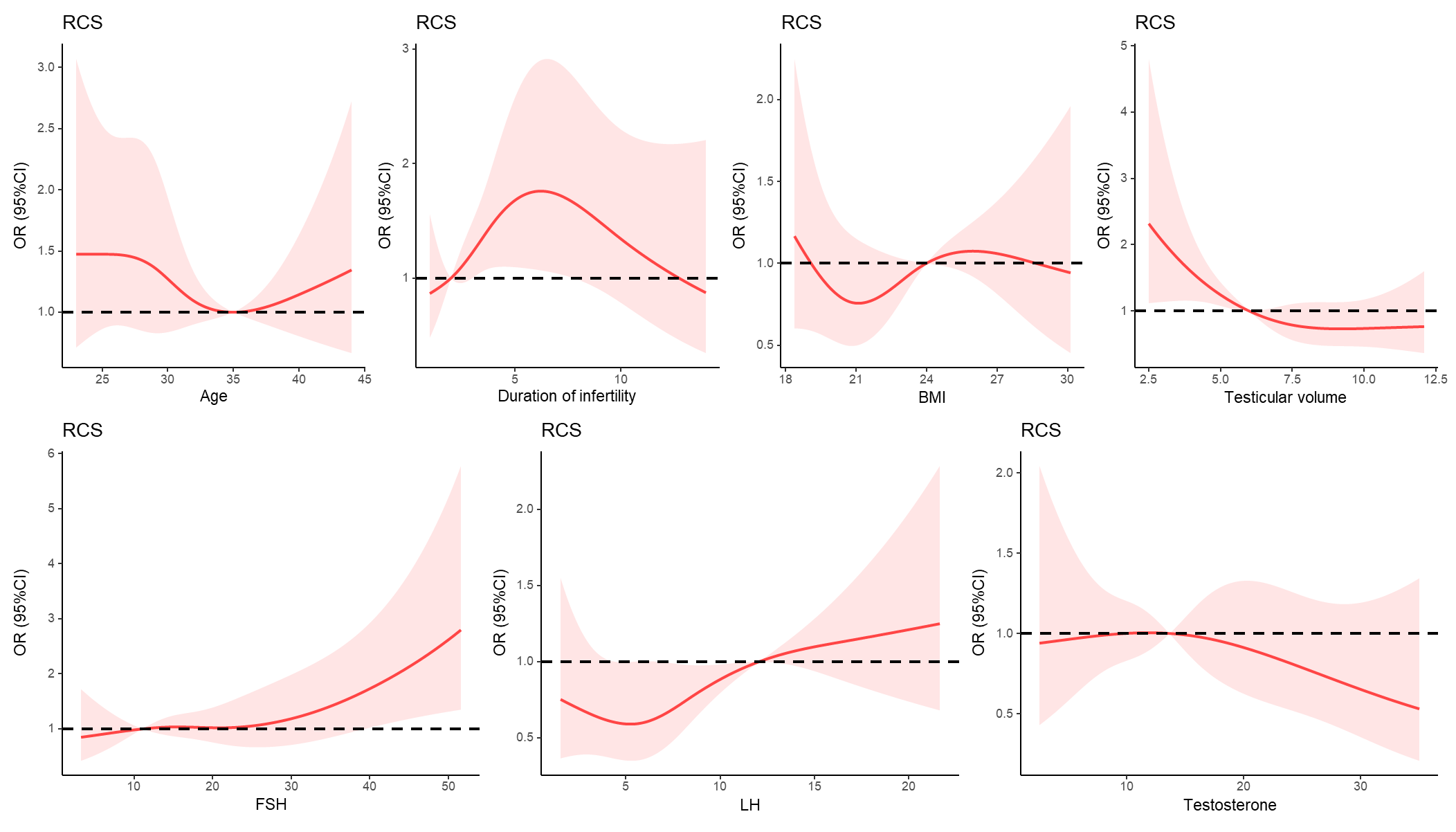


**Supplemental Fig. 4** Association of clinical characteristics

with SRR in NOA men.

Supplement: Supplementary file 4 — Additional file 4: Supplemental Fig. 4. Association of clinical characteristics with SRR in NOA men. [file 12958_2022_934_MOESM4_ESM.docx]

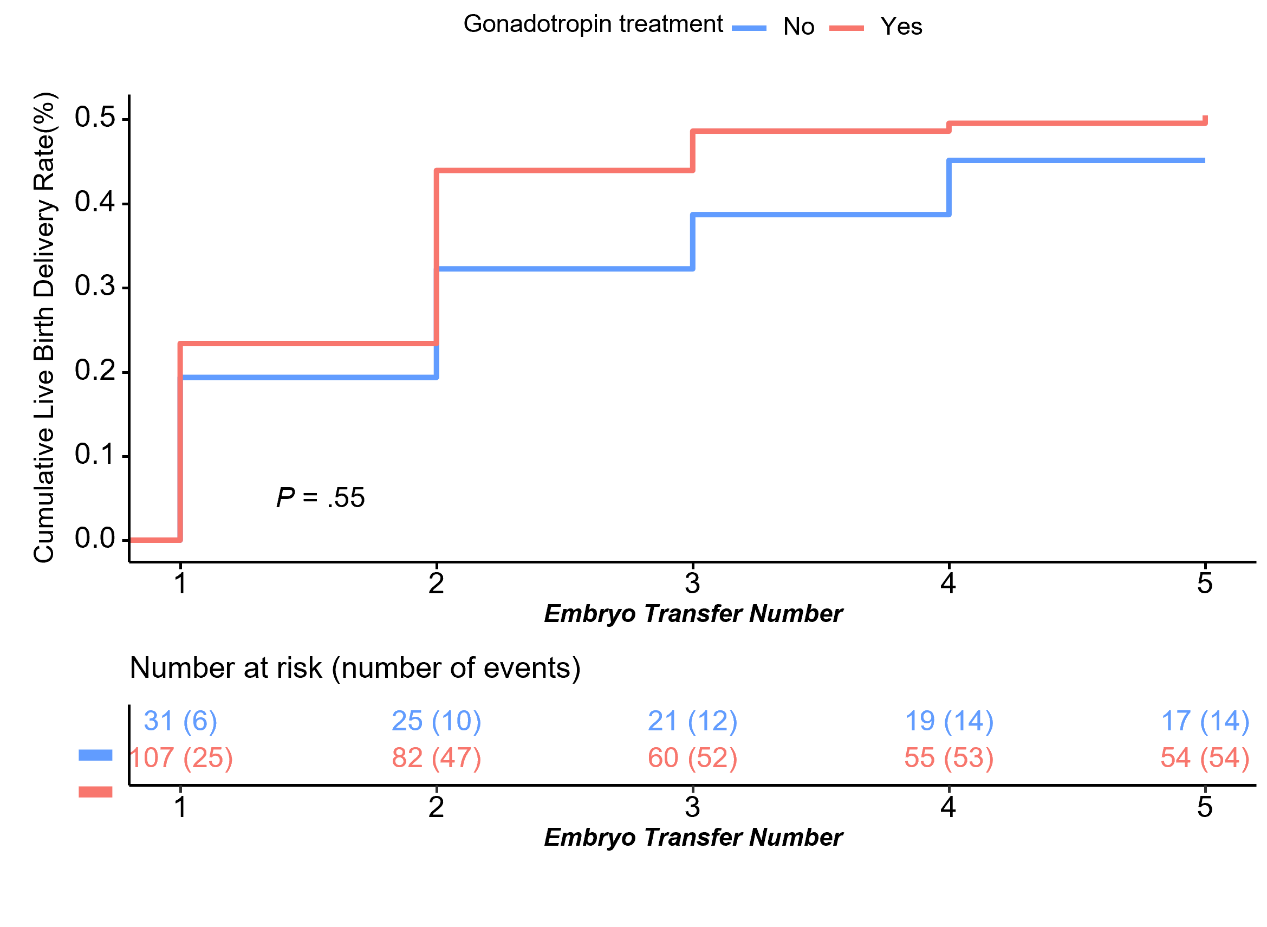


**Supplemental Fig. 6** Cumulative delivery plots and their correspondent tables.

Supplement: Supplementary file 6 — Additional file 6: Supplemental Fig. 6. Cumulative delivery plots and their correspondent tables. [file 12958_2022_934_MOESM6_ESM.docx]
