# Supplementary material for: Gonadotropins treatment prior to microdissection testicular sperm extraction in non-obstructive azoospermia: a single-center cohort study
Source: Reprod Biol Endocrinol. 2022 Apr 1;20:61. doi: 10.1186/s12958-022-00934-1 (PMC8973804; doi:10.1186/s12958-022-00934-1)
Supplement: Supplementary file 5 — Additional file 5: Supplemental Fig. 5. Causal mediation analysis for gonadotropin therapy. [file 12958_2022_934_MOESM5_ESM.docx]

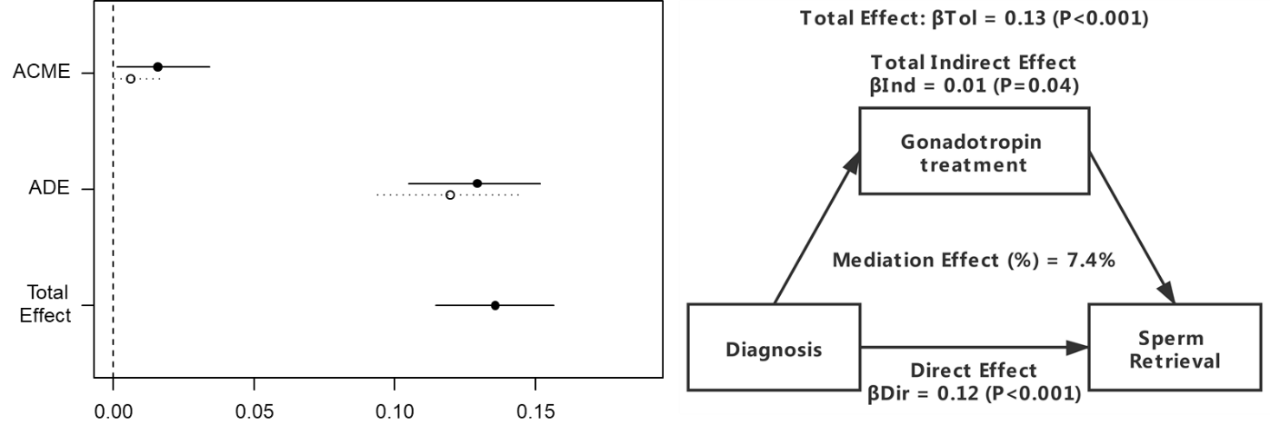


**Supplemental Fig. 5** Causal mediation analysis for gonadotropin therapy. The solid line represents the non-idiopathic NOA men, and the dashed line represents the idiopathic NOA.

Abbreviations: ACME: Average causal mediation effects; ADE: Average direct effects. β_Tot_: Total effect; β_Ind_: Total indirect; β_Dir_: Direct effect.
