# Supplementary material for: Gonadotropins treatment prior to microdissection testicular sperm extraction in non-obstructive azoospermia: a single-center cohort study
Source: Reprod Biol Endocrinol. 2022 Apr 1;20:61. doi: 10.1186/s12958-022-00934-1 (PMC8973804; doi:10.1186/s12958-022-00934-1)
Supplement: Supplementary file 7 — Additional file 7: Supplemental Table 1. Characteristics of NOA men receiving or not receiving gonadotropins therapy after the propensity score match. [file 12958_2022_934_MOESM7_ESM.docx]

**Supplemental Table 1.** Characteristics of NOA men receiving or not receiving gonadotropins therapy after the propensity score match

| Characteristic | Overall  (N=348) | No GN treatment  (N=174) | GN treatment  (N=174) | *P* value |
| --- | --- | --- | --- | --- |
| Age (years) (median [IQR]) | 31.00 [28.00, 35.00] | 32.00 [28.00, 35.00] | 30.00 [28.00, 34.00] | 0.078 |
| Duration of infertility, (years) (median [IQR]) | 3.00 [2.00, 5.25] | 3.00 [2.00, 5.00] | 3.00 [2.00, 5.75] | 0.969 |
| BMI (kg/m^2^) (median [IQR]) *^a^* | 22.00 [20.80, 25.00] | 21.80 [20.80, 24.30] | 22.50 [20.80, 25.60] | 0.225 |
| Bilateral testicular volume (mL) (median [IQR]) | 6.20 [5.10, 7.90] | 6.10 [5.10, 7.80] | 6.25 [5.20, 8.10] | 0.165 |
| Diagnosis (%) |  |  |  | 0.256 |
| Idiopathic | 277 (79.6%) | 146 (83.9%) | 131 (75.3%) |  |
| Cryptorchidism | 21 (6.0%) | 8 (4.6%) | 13 (7.5%) |  |
| Previous mumps and bilateral orchitis | 26 (7.5%) | 10 (5.7%) | 16 (9.2%) |  |
| AZFc microdeletion | 24 (6.9%) | 10 (5.7%) | 14 (8.0%) |  |
| Baseline hormone levels (median [IQR]) *^b^* |  |  |  |  |
| LH (μ/L) | 6.88 [4.86, 9.51] | 7.06 [4.70, 10.18] | 6.69 [4.92, 8.84] | 0.433 |
| FSH (μ/L) | 17.37 [12.35, 23.75] | 18.22 [12.39, 24.98] | 16.70 [12.34, 23.27] | 0.345 |
| T (nmol/L) | 13.90 [9.05, 19.50] | 13.77 [8.92, 19.70] | 13.96 [9.43, 19.20] | 0.991 |
| E_2_ (pmol/L) | 81.00 [60.00, 111.00] | 80.50 [58.00, 110.50] | 82.00 [62.00, 111.00] | 0.550 |
| PRL (ng/ml) | 9.73 [7.31, 13.13] | 9.22 [7.07, 13.00] | 10.58 [7.91, 13.28] | 0.054 |
| Sperm retrieval (%) | 85 (24.4%) | 34 (19.5%) | 115 (29.3%) | **0.046** |

Abbreviations: GN: Gonadotropins; BMI: Body mass index; FSH: Follicle-stimulating hormone; LH: Luteinizing hormone; PRL: Prolactin; IQR: Interquartile range.

Multiple imputation was used to account for missing data in the propensity score matched analysis.
