# Supplementary material for: Gonadotropins treatment prior to microdissection testicular sperm extraction in non-obstructive azoospermia: a single-center cohort study
Source: Reprod Biol Endocrinol. 2022 Apr 1;20:61. doi: 10.1186/s12958-022-00934-1 (PMC8973804; doi:10.1186/s12958-022-00934-1)
Supplement: Supplementary file 8 — Additional file 8: Supplemental Table 2. ICSI outcomes of couples stratified according to preoperative gonadotropin therapy and non-gonadotropin therapy. [file 12958_2022_934_MOESM8_ESM.docx]

**Supplemental Table 2.** ICSI outcomes of couples stratified according to preoperative gonadotropin therapy and non-gonadotropin therapy

|  | No GN treatment  (N=31) | GN treatment  (N=107) | *P*-value |
| --- | --- | --- | --- |
| ICSI cycles (n) | 57 | 188 | NA |
| Transferred cycles (n) | 35 | 124 | NA |
| Oocytes retrieved | 437 | 1646 | NA |
| Fertilization rate *^a^* (n/total) | 69.6% (257/369) | 62.5% (880/1407) | .014 |
| 2PN cleavage rate *^a^* (n/total) | 88.3% (227/257) | 84.0% (739/880) | .106 |
| D3 available embryos *^a^* (n/total) | 49.3% (112/227) | 49.7% (367/739) | .993 |
| Blastocyst rate *^a^* (n/total) | 32.3% (43/133) | 29.8% (134/450) | .649 |
| β-hCG positive rate *^a^* (n/total) | 68.6% (24/35) | 54.0% (67/124) | .180 |
| Clinical pregnancy rate *^a^* (n/total) | 54.3% (19/35) | 50.0% (62/124) | .798 |
| Live birth delivery rate *^a^* (n/total) | 45.2% (14/31) | 50.5% (54/107) | .752 |
| Miscarriage rate *^b^* (n/total) | 26.3% (5/19) | 12.9% (8/62) | .172 |

Abbreviation: NA: Not applicable.

Note:

*^a^* *P*-value was calculated using the Chi-squared test.

*^b^* *P*-value was calculated using Fisher’s exact test.
